# Surgical treatment strategies for hepatic alveolar echinococcosis

**DOI:** 10.1016/j.fawpar.2019.e00050

**Published:** 2019-04-05

**Authors:** L.A. Salm, A. Lachenmayer, S.F. Perrodin, D. Candinas, G. Beldi

**Affiliations:** Department of Visceral Surgery and Medicine, University Hospital Bern, Bern, Switzerland

**Keywords:** Alveolar echinococcosis, *Echinococcus multilocularis*, Resection, Surgical strategies, Interventional treatment, Liver transplantation

## Abstract

Alveolar echinococcosis is a severe and rare helminthic disease with increasing incidence in endemic regions. Herein, available evidence on curative surgical and potential palliative approaches was reviewed. Such strategies have to be applied in the context of available resources in different health-care systems. Complete resection followed by adjuvant therapy remains the only curative treatment available. Curative surgery is performed by open or laparoscopic approach depending on the extent of the disease and the experience of the surgical team. Palliative resections are typically not indicated, because the availability of endoscopic treatments of biliary complications and long-term benzimidazoles represent efficient alternatives to surgery. Liver transplantation as an alternative to palliative surgery has not been shown to be superior to long-term conservative therapy. Immunosuppressive therapy might additionally contribute to fatal disease recurrence after transplantation.

## Highlights

• Alveolar echinococcosis is an aggressive zoonotic infection caused by the parasite *Echinococcus multilocularis.*
• Surgery remains the only curative treatment strategy for alveolar echinococcosis.
• In non-resectable patients, benzimidazole treatment is preferred over palliative surgery or liver transplantation.
• Follow-up is based on annual serological testing using Em18-Antigen and cross-sectional imaging.

## 1 Introduction

Alveolar Echinococcosis (AE) is an aggressive and potentially lethal zoonotic infection, and the number of reported cases of AE are increasing in endemic regions (Eckert and Deplazes, 2004). Humans are infected by the larval stages (metacestodes) of *Echinococcus multilocularis* tapeworms and act as aberrant hosts (Eckert and Deplazes, 2004). Due to the locally invasive growth pattern and potential to develop metastases, AE is often considered a tumor-like disease (Kern et al., 2017). Although AE predominantly occurs in the liver, in 3% of cases other organs such as the diaphragm, peri-renal tissue, lymph nodes, peritoneum, lung, brain, spleen or bone are infected as well (Sarwari, 2018). While one-third of the cases are detected incidentally, some patients may present with abdominal pain or jaundice (Brunetti et al., 2010). Infections become clinically apparent after long incubation periods of 5–15 years (Brunetti et al., 2010). According to the World Health Organization (WHO) guidelines, the diagnosis of AE requires at least one of the following four diagnostic criteria: 1) typical organ lesion in radiological examination (abdominal ultrasound, computed tomography (CT) or magnetic resonance tomography (MRT)), 2) detection of *Echinococcus* spp. specific serum antibodies 3) detection of parasitic vesicles and laminated layer in histopathology 4) detection of *E. multilocularis* nucleic acid sequences (Brunetti et al., 2010). Among these four dimensions for detection, immunodiagnosis of AE is an important tool for the specific and early detection of the infection. *E. multilocularis* antigens (Em2, Em2+, Em18) have been widely evaluated and used in enzyme-linked immunosorbent assay (ELISA) and show a high diagnostic sensitivity of 90–100%, with a specificity of 95–100% (Brunetti et al., 2010; Gottstein et al., 1993; Sarwari, 2018).

Without adequate treatment, AE grows slowly and progressively in the liver, which is why a treatment with curative intent should be attempted whenever possible. While complete resections are associated with good long-term results (Sarwari, 2018), the prognosis of patients who receive reductive or debulking procedure is poor (Kasai et al., 1980; Kawamura et al., 2011). Unfortunately, most patients in the last century were diagnosed at advanced disease stages with no curative treatment options available (Kadry et al., 2005). Only since the introduction of benzimidazoles (BZM) in the 1970s the prognosis of AE could be drastically improved, achieving survival rates almost similar to that of healthy controls (Buttenschoen et al., 2009; Kadry et al., 2005; Torgerson et al., 2008). In this review, the evidence and results of surgical strategies are reported and include the technical developments in hepatobiliary surgery such as laparoscopic liver resection, percutaneous or open ablation and auto- and orthotopic liver transplantation. Furthermore, the relevant role of minimal-invasive treatments of biliary complications and medical therapy with BZM to improve the outcome of non-resectable patients is presented.

## 2 Surgery for AE

### 2.1 Curative versus palliative resection

Whenever technically feasible, radical surgical resection of the entire parasitic mass is the only curative therapeutic option for AE (Brunetti et al., 2010; Buttenschoen et al., 2009; Chen et al., 2018; Du et al., 2016; Eckert and Deplazes, 2004; Hillenbrand et al., 2017; Joliat et al., 2015; Kadry et al., 2005; Kawamura et al., 2011). Complete resection is usually defined as complete resection of the lesions confirmed by pathological specimens (Joliat et al., 2015; Kawamura et al., 2011; Hillenbrand et al., 2017). We analyzed studies reporting resection of AE between 1972 and 2016 in this review (Table 1). The included studies mostly originated from high-income settings, with good quality of medical infrastructure and accessibility to health services. Case-reports and case-series were excluded. Over time, strategies changed from a relatively high rate of reductive to more curative surgeries in recent years. While most authors showed no benefit of reductive surgery compared to medical treatment alone, some even reported a worse survival due to a higher rate of disease progression and complications (Buttenschoen et al., 2009; Chen et al., 2018; Frei et al., 2014; Kadry et al., 2005). Furthermore these studies were limited because results of reductive surgery were compared with historical controls or other published series. Therefore, any potential benefits of palliative resections are likely to be overestimated. Parasite size and invasion of hepatic blood vessels or adjacent organs were significantly correlated with the inability to perform a complete resection (Kawamura et al., 2011). Based on this existing evidence, the WHO recommends to avoid reductive resections for AE and to perform curative resections whenever possible (Brunetti et al., 2010). The average rate of curative resection is 63% (21–87%) (Table 1). The rate of curative resection was increasing from 21% in patients surgically treated between 1982 and 1999 to 87% after 2000 (Buttenschoen et al., 2009). Procedures in the more recent time period were increasingly performed for early stage AE, suggesting that the use of modern imaging modalities such as CT and MRT led to diagnosis and treatment at an earlier stage. Additionally, the overall progress in hepatobiliary surgery has most probably contributed to the higher rates of complete resection in AE patients.

**Table 1:** patient characteristics and demographics of eligible studies

Author (year of publication)	Number of patients (n)	Subgroups	Inclusion time frame	Mean patient age (years)	Mean lesion-size (cm)	Curative resection rate	Reductive resection rate	Other treatment^a^	Safety margin	Morbidity/Mortality^b^	Recurrence rate	Overall survival (%, years of follow-up)
Kadry et al. (2005)	113	Gr A: complete resection Gr. B: reductive surgery Gr C: BZM alone	1976 - 2003	52	NR	64.1%	21.2%	40.7%	NR	NR	NR	NR
Buttenschoen et al. (2009)	36	Gr. A: 1982-1999 Gr B: 2000-2006	Gr. A: 1982-1999 Gr. B: 2000-2006	NR	NR	Gr A: 21% Gr B: 87%	Gr A: 79% Gr B: 14%	0%	Gr. B: 20 mm	Morbidity: 15% Hospital lethality: 3%	Curative resection: 11% (2/18)	Curative resection: 100% Reductive surgery: 50%
Kawamura et al. (2011)	188	Gr A: complete resection Gr. B: reductive surgery Gr. C: drainage or laparotomy	1984-2009	Gr. A : 53.1 Gr. B : 51.6 Gr. C : 60.6	Gr A : 5.1 Gr B : 9.3 Gr C : 15	63.5%	33.5%	3%	NR	Mortality: 4.2%	Gr. A: 3.5% (10y), 5.6% (15y, 20y) Gr. B: 12.9% (10y), 28.4% (15y), 38.6 % (20y) Gr.C : 50.0% (10y), 66.7% (15y)	Gr A: 98.9% (10-15-20y) Gr B: 97.1% (10y), 92.8% (15y), 61.9% (20y). Gr C : 50.0% (10y), 33.3% (15y)
Joliat et al. (2015)	59	No subgroups	1992-2013	60	5.5	71%	29%	0%	NR	Complication: 36% (n=21) Dindo I-II: 25% (n=15) Dindo III-IV: 9% (n=5) Dindo V: 2% (n=1) Mortality: 3%	Complete resection: 2% (n=1) Reductive surgery: 41% (n=7)	97%
Du et al. (2016)	144	Gr. A: complete resection Gr. B: reductive surgery	2004-2015	38.4	Gr A : 8.1 Gr B: 12.5	58.3%	41.6%	0%	NR	Complication: 10.4% Dindo I-II: 5.5% Dindo III-IV: 2.7% Dindo V: 2.05%	NR	NR
Hillenbrand et al. (2017)	92	No subgroups	1993-2003	40.5	NR	NR	NR	NR	< 1 mm >20 mm	NR	16%	NR
Chen et al. (2018)	115	Gr. A: radical resection Gr. B: reductive resection Gr C: liver transplantation	2004-2016	47.8	NR	66.9%	14.7%	18.4%	NR	NR	NR	Gr. A: 97% Gr. B: 70.6% Gr. C: 81%

Gr: Group: NR: not reported, BZM: Benzimidazole.

a Other treatment include BZM only therapy (Kadry et al., 2005), liver transplantation (Chen et al., 2018), drainage or laparotomy (Kawamura et al., 2011).

b Morbidity and mortality are defined according to the Dindo-Clavien classification (Dindo et al., 2004).

AE typically grows along the biliary tract and may therefore infiltrate the liver diffusely beyond the primary mass (Joliat et al., 2015; Ozturk et al., 2009). Thereby, early recurrences are still reported after resections with a curative intent and are likely to be associated with macroscopically undetectable residual parasite in the liver (Buttenschoen et al., 2009). However, recurrences within one year of curative resection are unusual due to the slow growth of AE (Hillenbrand et al., 2018).

While the WHO guidelines recommend to perform radical resections of the entire parasitic mass as a curative treatment, it is unclear if this approach should include extensive surgery with resection of adjacent organs or distant metastases (Joliat et al., 2015). Some studies recommend to combine liver resection with resection of extrahepatic disease including metastases or infiltrated adjacent organs (diaphragm, adrenal gland, lung, pancreas, stomach) (Hillenbrand et al., 2017). However, a small wedge resection of the diaphragm or infiltrated stomach cannot be compared to extensive multi-visceral or even thoraco-abdominal resections requiring long surgeries with high complication rates. Recently, a case series suggested extended hepatectomy with autotransplantation as a therapeutic alternative for patients with advanced AE (Aji et al., 2018). However, the reported 30-day mortality of 7.24% and overall mortality (>90 day) of 11.5% was exceedingly high, respectively, with a median follow up of only 22.5 months (Aji et al., 2018). Furthermore, no information was given on the availability and duration of BZM therapy in this study which might have been a valid treatment alternative with a satisfying outcome. Such poor outcome is clearly worse than what is observed with conservative approaches in European centers (Beldi et al., 2019).Therefore any indication for such an extensive surgical treatment of AE with high morbidity and mortality should be critically questioned with regard to the satisfying results of conservative life-long medical treatment with BZM. However, AE is widespread in countries with limited medical resources (Li et al., 2010; Usubalieva et al., 2013), where access to BZM therapy and availability of follow-up regimens are limited. Under such circumstances extensive surgical therapy might be the only option.

### 2.2 Role of laparoscopy for AE resection

Even though laparoscopic liver surgery is increasingly used for oncologic liver resections, little is published with regard to this approach in the treatment of AE. However, first results are promising (Kawamura et al., 2011). This observation is in line with our own experience with laparoscopic resections of AE showing no difference in efficacy and safety compared to open AE resections or laparoscopic surgeries for other liver malignancies (unpublished data). Although AE might be a good indication for laparoscopic resection whenever technically feasible, larger studies are clearly needed to validate these initial results.

### 2.3 Anatomical versus atypical resection

Most of the current literature on surgical treatment of AE does not specify the exact type of resection. In contrast to oncologic tumor resections, where anatomical resections (complete resection of functional parenchyma areas) and atypical resections (resections without consideration of functional boundaries) have been compared and discussed (Li et al., 2017), no detailed data is available for the resection of AE. Almost 50% of the 643 reported resections of the 7 studies summarized in Table 1 were classified as major anatomical liver resections, with 17% right hemihepatectomies, 9% extended right hemihepatectomies, 10.4% left hemihepatectomies and 6% extended left hemihepatectomies. A segmentectomy (resection of at least one segment) was performed in 26% of patients. Atypical resections were also successfully performed in some of the studies for smaller lesions (Buttenschoen et al., 2009; Hillenbrand et al., 2017; Kadry et al., 2005; Kawamura et al., 2011). However, neither the exact number of resected segments nor comparisons of the different procedures or correlations with outcome have been reported so far.

### 2.4 Resection margin

The role of the resection margin has been analyzed and seems to be significantly correlated with local recurrence and survival. Since the germinal layer of AE infiltrates adjacent tissue and the safety margin may be difficult to assess radiologically prior to surgery or macroscopically during the procedure (Eckert et al., 1983), experts recommend a 20 mm safety margin to avoid local recurrence (Brunetti et al., 2010). However, there is little data supporting this recommendation. Table 1 summarizes the different safety margins used in the different studies. The reported safety margins ranged between 1 mm and 20 mm. A resection margin of 20 mm may be difficult to obtain due to the frequent close proximity of important central liver vasculature and bile ducts (Hillenbrand et al., 2017). Nevertheless, a safe distance of at least 1 mm, in combination with adjuvant BZM therapy for two years, resulted in long-term disease-free survival (Hillenbrand et al., 2017). Of the 15 (16.3%) patients that presented with a local recurrence in their study, 13 (86.7%) had a safety distance of <1 mm, and two had 1 mm and 3 mm, respectively (Hillenbrand et al., 2017). In other words, a recurrence occurred in only two out of 59 patients (3.8%) with a safety distance of 1 mm or more (Hillenbrand et al., 2017). Future studies should incorporate the extent of disease, as it is a relevant confounding factor, as well as the indication for surgery in the light of applied conservative treatment strategies. Furthermore, small particles of *E. multilocularis* (SPEMs) have been detected up to 1.5 mm away from the main lesion as well as in lymphoid aggregates (Barth et al., 2012). However, it remains unclear if these SPEMs are relevant for the definition of the resection status.

### 2.5 Role of liver transplantation in treatment of AE

Due to the rarity of the disease and the excellent outcome of patients undergoing complete resection, as well as acceptable results after conservative treatment, there is only limited published data on liver transplantation (LT) in patients with AE, with few small and retrospective analyses. The WHO currently still recommends to consider transplantation if all of the following findings are present: 1) severe liver insufficiency or recurrent life-threatening cholangitis, 2) a radical liver resection cannot be performed, and 3) absence of extra-hepatic disease (Brunetti et al., 2010). However, in the light of the satisfying long-term results with conservative therapy, such strategies have to be regarded with caution. The overall survival rate (OS) after LT is rather poor, with 71–85% at 1 year, 71% at 5 years and 49% at 10 years (Ambregna et al., 2017, Bresson-Hadni et al., 1999, Koch et al., 2003, Pan et al., 2004, Xia et al., 2005). Mortality rates after LT seem to be higher than after resection, with studies reporting a mortality rate of 25–45% after liver transplantation, including some perioperative deaths (Koch et al., 2003; Pan et al., 2004). Patients included in these studies were often transplanted for advanced stages with multiple biliary and infectious complications. In addition, early disease recurrence after transplantation significantly hampered the outcome (Joliat et al., 2015; Koch et al., 2003). The authors of the largest study, which retrospectively analyzed 45 cases of transplantations for AE, acknowledged the high recurrence rates because of difficult hepatectomies due to enormous parasitic masses infiltrating blood vessels, bile ducts and other adjacent organs, and the immunosuppressive therapy (Koch et al., 2003). The complication rate is significant as 34% patients after LT suffered major complications including perihepatic clotting, hepatic artery thrombosis, hepatic artery folding, diaphragmatic rupture or postsurgical liver infection (Koch et al., 2003). Furthermore, four patients had to undergo a retransplantation, because of primary nonfunction of the liver, chronic rejection or chronic cholangitis. Bresson-Hadni et al. (1999) confirmed the latter observation in a long-term evaluation of 15 patients after LT showing an elevated risk of AE recurrence (Bresson-Hadni et al., 1999). Immunosuppression entails the risk of re-growth of larval remnants and the formation or growth of metastases, particularly in the brain (Bresson-Hadni et al., 1999; Koch et al., 2003; Vuitton et al., 2006). BZM therapy is generally indicated preoperatively to stabilize the progression of the disease, and postoperatively to avoid recurrences (Bresson-Hadni et al., 2003). Therefore, LT should not be performed in patients who do not tolerate BZM therapy. In conclusion, LT should be reserved for special, highly selected cases, and used as a salvage therapy only (Beldi et al., 2019).

### 2.6 Role of thermal ablation for treatment of AE

Percutaneous ultrasound-guided microwave ablation (MWA) was reported in the literature as a novel and effective therapeutic method for single AE lesions under 5 cm in diameter (Cairang et al., 2017). In this study, 12 patients were assessed retrospectively, and all patients showed complete ablation in post-interventional CT (Cairang et al., 2017). No serious complications occurred as result of the MWA (Cairang et al., 2017). Our own experience revealed that because of the dense fibrous capsule, AE lesions may be very difficult to penetrate and therefore may not be suitable for percutaneous ablation (unpublished results). Furthermore, calcifications of the lesions might affect treatment efficacy in these patients. Thus, multicentric prospective studies are necessary to determine the value of MWA in treatment of AE.

### 2.7 Complications after surgical treatment of AE

Complications after AE resection are similar to those of general liver surgery and highly depend on the extent of resection performed. A Japanese study investigated risk factors for the development of complications after hepatic resections in 793 patients and described surgery duration >360 min, blood loss >400 mL, and serum albumin level < 3.5 g/dl as independent risk factors for morbidity in the multivariate analyses (Kamiyama et al., 2010). Depending on the extent of liver surgery, reported complication rates in patients with AE varied between 15% and 36%, and mortality rates after AE resection were between 3% and 4.2% in the studies summarized in Table 1.

## 3 Interventional management of biliary complications of AE

The treatment of biliary complications of AE including cholangitis, obstructive jaundice, abscess and biliary fistula, is often challenging due to parasitic invasion of the biliary tree (Ozturk et al., 2009; Sezgin et al., 2005). However, several endoscopic procedures can be performed prior to surgery or as a palliative treatment in patients with non-resectable disease (Joliat et al., 2015). According to the WHO guidelines, endoscopic and percutaneous interventions are indicated for biliary complications in non-resectable patients (Brunetti et al., 2010). In the last decades, percutaneous and endoscopic bile or abscess drainage have replaced palliative surgery with jejuno-biliary anastomosis to treat biliary complications (Bresson-Hadni et al., 2006; Ozturk et al., 2009; Sezgin et al., 2005). Compared to percutaneous drainage, endoscopy has the advantage of avoiding a permanent external drainage through an internal drainage (Bresson-Hadni et al., 2006). Endoscopic sphincterotomy and insertion of several plastic stents for structural changes (stenosis, distorsion and obstruction) of the external biliary tract improve biliary drainage and treat cholestasis (Ambregna et al., 2017; Ozturk et al., 2009).

## 4 BZM therapy

### 4.1 Conservative treatment

The WHO guidelines recommend albendazole (ABZ) or mebendazole (MBZ) as the treatment of choice for AE (Brunetti et al., 2010). Both ABZ and the older compound MBZ are known for their high therapeutic efficacy and have similar response rates (Reuter et al., 2000). However, BZM are only parasitostatic, i.e. growth of the parasite is stopped without killing the parasite (Reuter et al., 2004). Treatment was simplified and costs were reduced by 40% with the introduction of the twice daily ABZ regimen (Reuter et al., 2000). Generally, BZM treatment is well tolerated and can be taken as a life-long medication (Brunetti et al., 2010; Reuter et al., 2000). Known side-effects include toxic hepatitis, neutropenia, reversible alopecia and vertigo (Kern, 2010; Reuter et al., 2000). Due to potential hepatotoxicity, monitoring of serum transaminases is necessary for the duration of BZM therapy (Reuter et al., 2000). Whether stricture of the hilar structures (portal vein and common bile duct) resulting in cholestasis and esophageal variceal bleeding are a late complication of long-term BZM therapy or the consequence of the primary disease remains unclear (Ammann et al., 1994).

### 4.2 Adjuvant treatment

Postoperative treatment with BZM is recommended for an additional 2 years after curative resection in order to prevent recurrence of the disease (Brunetti et al., 2010; Reuter et al., 2000). This recommendation is based on a small observational study and a review of the literature by Reuter et al. (2000) showing that treatment with BZM is effective and safe (Reuter et al., 2000). To our knowledge there is no study comparing curative resection with adjuvant BZM therapy and curative resection without adjuvant BZM therapy. In most studies, BZM therapy was continued for 2 years after curative resection, and life-long BZM therapy was administered after reductive surgery (Buttenschoen et al., 2009; Du et al., 2016; Joliat et al., 2015; Kadry et al., 2005). However, alternative postoperative drug regimens have also been described. The drug regimen varied from no postoperative BZM-treatment to more than two years BZM-treatment in one study (Hillenbrand et al., 2017). In another study, the data on BZM therapy was completely missing (Chen et al., 2018). The different outcomes between the groups with and without BZM therapy after curative resections have not been analyzed so far. However, in patients that underwent resection without a safety margin, AE seems to reoccur after the adjuvant BZM therapy was stopped, leading to late recurrences after up to 24 years (Hillenbrand et al., 2017). The authors concluded that after resection with at safety margin of at least 1 mm, BZM therapy for two years might offer a significant chance of remaining disease-free on the long term (Hillenbrand et al., 2017). Thus, because of the lack of data, patients with incomplete resection likely need a life-long adjuvant therapy to prevent disease progression and recurrence. However, for complete resection, evidence for or against adjuvant therapy is poor, irrespective of the resection margin. We propose adjuvant BZM therapy for 2 years, with annual follow-up consisting of imaging and serology (Sarwari, 2018). In patients with wide resection margins and no detectable antibodies against Em18 antigen, BZM may potentially be stopped earlier. In patients with LT, BZM should be given long-term because of the risk of opportunistic AE infections or recurrence due to immunosuppression.

### 4.3 Neoadjuvant (i.e. pre-surgery) treatment

Unlike for cystic echinococcosis, not much is known about the role of neoadjuvant treatment regimen, although most patients receive BZM therapy before surgery. Furthermore, there is no data on the duration of pre-surgery treatment (Hillenbrand et al., 2017; Joliat et al., 2015; Kadry et al., 2005; Kawamura et al., 2011). Even though the WHO Guidelines clearly recommend a pre-surgical BZM treatment before LT (Brunetti et al., 2010), one multicenter study showed that only 58% of patients received a neoadjuvant treatment with BZM (Koch et al., 2003).

## 5 Outcome after surgical treatment of AE

### 5.1 Survival

The largest study on surgical therapy for AE is from Japan and showed that a complete resection of the parasite in combination with adjuvant therapy with BZM resulted in almost 100% survival after 20 years (Kawamura et al., 2011). However, only 63.3% of patients of this study were suitable for a complete resection due to the advanced stage of the disease at diagnosis (Kawamura et al., 2011). The OS after reductive surgery is reported to be between 50% and 97% depending on the follow-up time (Table 1) (Kawamura et al., 2011). Using univariate analyses, the parasite size (>9 cm), hepatic vein invasion, portal vein invasion, lung metastasis and curability were identified as major risk factors that negatively affected OS. However, only curability, which is defined as complete resection, was identified as an independent risk factor in multivariate analysis (Kawamura et al., 2011). The development of biliary complications in patients with non-resectable AE requiring long-term BZM occurred in 28% (26/148) of patients and was associated with a median survival of 3 years only (Frei et al., 2014). A multicenter study showed a survival rate after LT for AE of 77%, 71% and 49% after 1, 5 and 10 years respectively (Koch et al., 2003).

### 5.2 Recurrence

Reported recurrence rates after curative resection are between 2% and 16% at 5–20 years (Table 1). Recurrence usually occurs at the site of resection, while the development of new intrahepatic lesions or lesions in the area of the hepatoduodenal ligament are rather uncommon (Hillenbrand et al., 2018; Hillenbrand et al., 2017). Because AE usually develops slowly, detection of recurrent disease within the first postoperative year is unusual (Hillenbrand et al., 2017). Although lymph node involvement is frequent in AE, a German study was able to show that lymph node involvement in contrast to most malignant tumors is not associated with recurrence (Hillenbrand et al., 2018). Recurrence after transplantation was reported to occur in 23% after 1 year, 34% after 3 years, 42% after 5 years and 55% after 10 years (Koch et al., 2003). In 6 of 45 (13%) patients a parasitic disease recurrence of the transplanted liver is reported (Koch et al., 2003).

## 6 Follow up after surgical treatment of AE

According to the WHO guidelines, long-term follow-up with ultrasound and a follow-up with MRI/CT every 2–3 years is recommended after initiation of any type of treatment in patients with AE (Brunetti et al., 2010; Buttenschoen et al., 2009; Hillenbrand et al., 2017). Due to the possibility of late recurrences, Hillenbrand et al. (2017) recommend a long-term follow up (Hillenbrand et al., 2017). While any enlargement of a lesion over time is documented as progression (Brunetti et al., 2010), metabolic activity can be measured in the fludeoxyglucose – positron-emission tomography (FDG-PET) (Reuter et al., 2004). Even though a study showed a sensitivity of 91% for the detection of an active lesion in the FDG-PET, the same authors reported that 18 months after discontinuation of BZM in patients with inoperable AE, 8 of initially 15 PET-negative patients showed either new activity on PET (n = 6) or signs of clinical progression (n = 2) (Reuter et al., 2004). None of the currently available imaging modalities is able to evaluate the disease activity on its own (Reuter et al., 2004; Tappe et al., 2009). Serologic analyses of circulating antibodies show a good correlation with disease activity and should therefore be used as follow-up after curative resection (Tappe et al., 2009). While a rapid and complete decrease of anti-Em18 antibodies has been observed after successful curative resection, it remains unclear how long patients should be serologically monitored after surgery (Tappe et al., 2009). In countries where there are no possibilities for serological or radiological follow-up, the duration of BZM therapy needs to be gauged by the extent of the disease during surgical resection. In patients with extensive disease long-term BZM therapy needs to be evaluated.

## 7 Conclusion

Although complete resection followed by adjuvant BZM therapy is the only curative treatment option so far, life-long BZM therapy alone also significantly improves survival. Due to the specific morphological characteristics and the rarity of the disease, treatment should ideally be performed in high-volume centers, offering the whole spectrum of conservative and surgical treatment including minimal-invasive procedures. Although larger studies are needed to confirm the data, the width of the safety margin seems to be less important than initially thought if patients receive adjuvant BZM therapy. LT and extensive surgery such as autotransplantation should be reserved for exceptional cases as life-long BZM generally represent a good alternative. After LT, life-long BZM therapy is required, but the duration of BZM in resected patients with minimal or no safety margins remains unclear. A long-term follow-up with serological tests and imaging of patients is clearly recommended; however, the best suited imaging method and frequency still need to be determined. Future studies should focus on the evaluation of the duration of adjuvant chemotherapy and personalized adaptation to the individual risk of recurrence. Furthermore, studies assessing best treatment options in resource-poor regions, where most of the global AE cases occur, are urgently needed.

## Declaration of interests

None.
